# Agreement between Framingham, IraPEN and non-laboratory WHO-EMR risk score calculators for cardiovascular risk prediction in a large Iranian population

**DOI:** 10.34172/jcvtr.2020.04

**Published:** 2019-12-30

**Authors:** Mohsen Mirzaei, Masoud Mirzaei

**Affiliations:** Yazd Cardiovascular Research Centre, Shahid Sadoughi University of Medical Sciences, Yazd, Iran

**Keywords:** Risk Assessment, Cardiovascular Diseases, Framingham Risk Score, Iran

## Abstract

***Introduction:*** Estimation of the risk of cardiovascular diseases (CVD), may lead to prophylactic therapies. This study aims to compare and evaluate the agreement between CVD prediction of Iran Package of Essential Non-communicable Disease (IraPEN) and Framingham risk score (FRS).

*** Methods:*** All 40-79 years old participants in the Yazd Health Study who did not have a history of CVD were included. The 10-years risk of CVD was estimated by the laboratory (IraPEN), non-laboratory WHO-EMR B and FRS. The risk was classified into low, moderate and high-risk groups. Cohen’s weighted kappa statistics were used to assess agreement between tools. To assess discrepancies McNemar’s χ2 test for paired data was used. P values < 0.05 were considered statistically significant.

***Results:*** In total, 2103 participant was included and the risk scores were calculated. Of them, 26.5% were stratified as high risk by FRS, compared with 6.1% by IraPEN. A slight agreement (37.9%) was observed (kappa 0.17, *P* < 0.0001), in other words. This discrepancy between IraPEN vs. FRS was seen in both sexes (*P* < 0.0001), although in women the agreement ratio was higher (52.1% vs. 21.3%). The discrepancy between FRS and IraPEN in categorizing people at risk of CVD was 55.5%, (*P* < 0.0001) but this was not significant between IraPEN and non-laboratory WHO-EMR-B (World Health Organization - Eastern Mediterranean Regional-B group countries) score (*P* < 0.523; discrepancies, 5.8%).

***Conclusion:*** Our study shows a slight agreement between various CVD risk scores. Thus, reviewing the IraPEN and using alternative tools for the low-risk group should be considered by decision-makers. It is important to use a more reliable score for nation-wide risk assessment.

## Introduction

Cardiovascular diseases (CVDs) are the most common cause of death in Iran and the world and contribute to disability and poor quality of life.^1-3^ Every year, 17.7 million of the world population (31%) die from CVD, of whom more than 75% occur in low-income countries.^4^

In Iran, unlike developed countries, death from CVD shows an increasing trend, probably because the advanced therapeutic methods have increased the patient’s life expectancy, however, the first occurrence of the disease is associated with high mortality.^5^ Hypertension, diabetes, obesity, tobacco smoking, and high serum cholesterol are among the major risk factors for CVDs, which, together with socioeconomic factors and people’s lifestyle (physical inactivity, poor diet, etc), have a synergistic effect on the disease incidence. These risk factors are modifiable and can be prevented and controlled by effective interventions.^6^ Implementing cost-effective interventions requires identification of high-risk groups at risk of CVD.

A cardiovascular risk score is a useful tool for calculating the probability of myocardial infarction (MI) in the next 5-10 years. With proper scoring, the primary health care resources can be targeted to the high-risk group that benefits from initiating and treating preventive interventions. The Framingham Heart Study helped identify high-risk individuals based on risk factors and select preventive interventions for each group, depending on the existing risk. Other commonly used risk scores include: Systematic COronary Risk Evaluation (SCORE) algorithm, QRISK, QRISK2 and WHO risk score.^7^

Using these tools, in one population may not be necessarily desirable in other communities with different ethnicity, culture and risk exposure duration, and lifestyle.^8^ Findings from studies in different regions have identified the preferences for each of the common risk-assessment tools for their populations. In India, Framingham risk score (FRS) is more suitable for preventive intervention,^9^ and FRS/SCORE found to be more appropriate for Malaysia.^10^ In countries with low resources, which do not have national cohort studies, WHO recommended a tool with two charts (Laboratory and non-laboratory) for prediction and prevention of CVDs.^11^

A comparison of different risk management tools can show the degree of agreement of tools in diverse populations. This is an incentive to persuade national health funding organizations to support longitudinal studies in order to design specific CVD prevention tools fine-tuned for their population.

By implementing a non-communicable diseases (NCDs) prevention and care program in Iran based on the WHO Package of Essential NCD interventions for primary health care (PEN); its risk model is recommended in the health system and integrated into the program of primary health care centers for the Iranian population (IraPEN).^12,13^ SIB (apple in Persian) is an integrated electronic health record system which is in use in Iran’s health system since 2016. Iranians’ health data are recorded there, and it generates health information by demand. Also, there is a possibility of a link with other registry systems. IraPEN used a risk scoring tool according to laboratory-based WHO risk score that assesses CVD risk of Individuals who referred to health centers to be evaluated for various risk factors. After entering their information on the SIB, the risk of 10 years CVD risk will be determined. Then, according to the risk level, colored cards are given as follows; green for low risk and yellow (10-19% risk), orange (20%-29% risk) and a red card for higher risk (≥30%). Self-care education is provided to all. Persons at risk of 20% or more are referred to the physician for further evaluation and intervention.^13^

In this study, we aimed to compare three cardiovascular risk prediction tools including WHO chart, Laboratory (IraPEN) and non-laboratory, and Framingham tool in a large Iranian population to assess the agreement between the model currently used in the health system of Iran. The results of this study can help policymakers to evaluate the accuracy of IraPEN tool for nationwide CVD risk score in order to redesign CVD preventive programs in the Iranian health system.

## Materials and Methods

This study is part of the enrollment phase of the Yazd Health Study (YaHS), which is a prospective cohort study on 10 000 residents of Yazd Greater Area. Details of the methodology have been published elsewhere.^14^ briefly; two hundred points were randomly selected according to postcodes. Each code was the starting point for a cluster. The interviewers then went to the neighborhoods of the first address, to interview 50 participants assigned to each cluster in equal numbers for men and women. Completion of questionnaire continued until the sample size was reached in each cluster. If a person was over the age of 69, s/he was also interviewed (n = 72). Furthermore, anthropometric and blood pressure measurements were carried out. An invitation card was sent to each participant to attend the laboratory. All measurements were performed on a standard laboratory protocol using Pars Azmoon kits and Ciba Corning (Ciba Corp., Switzerland) auto-analyzer.

Participants of both sexes aged between 40 and 79 years (age range common for both scores) with no history of CVD (i.e., acute MI, stroke, heart failure or chronic heart disease), Who carried out biochemical tests, were included. Persons with CVD history, out of the age range, or with missing information were not included in the study.

Ten-year cardiovascular risk assessment models; Framingham,^15^ IraPEN ^13^ and WHO-EMR B (non-laboratory) based on the WHO/ISH^16^ model were evaluated. In the Framingham model, age, diabetes, tobacco; treated/untreated systolic blood pressure and body mass index (BMI) in both sexes were considered as risk factors. The agreement of this tool with the laboratory base model, in which total cholesterol and HDL is used instead of BMI), was evaluated and, both models were found reliable for risk assessment.^17^ The 10-years cardiovascular risk was estimated by using MS Excel which is available at Framingham Heart Study website.^18^

In the IraPEN model, a risk map for the WHO-EMR B region (laboratory base) has been used to compare the assessment.^19^ in this model, the included risk factors were: age, diabetes mellitus, tobacco smoking, systolic blood pressure and total cholesterol in both sexes. In the WHO-EMR B risk score (non-laboratory), for individuals whose cholesterol is not measured, a separate chart is proposed that is also suitable for CVD risk.^20^ The 10-years risk of CVD in all models was classified into low, moderate and high-risk groups.^18,19^

All measurements were expressed as mean ± standard deviation (SD) or percentages for categorical variables. A descriptive analysis was conducted for basic characteristics in the studied population. The agreement between categorized risk estimates was assessed using Cohen’s weighted kappa statistic, presented with its 95% confidence interval (CI). According to Landis and Rock’s suggestion, the agreement ranges from −1 to 1 (with a perfect agreement=1). The following levels are often considered appropriate for judging. Poor if kappa < 0.00, slight; 0-0.20, fair; 0.21-0.40, moderate; 0.41-0.60, substantial; 0.61-0.80 and almost perfect; 0.80-0.99.^20^ To assess discrepancies, McNemar’s χ2 test for paired data (low and moderate/high risk) was used. P values < 0.05 were considered statistically significant. Analyses were performed using SPSS for Windows (version 16.0; SPSS Inc., Chicago, IL, USA).

## Results

A total of 2103 (35.4%) participants of 5945 participants, whose age range was 40-79 years, were selected for risk assessment by the designated models. The following participants were excluded: self-reported CVD (996 participants), who did not have biochemical test results (2846 participants). Of the total population analyzed, 45.9% (965) were men; 90.3% (1837) were native Yazdi population; 10.9% (230) were tobacco smokers, 25.8% (535) had a history of hypertension; 20.3% (424) were diabetics, and 24.7% (515) had a diagnosis of hypercholesterolemia. Mean fasting blood sugar (FBS) was 112.7 mg/dL (±4.2). Table 1 shows the characteristics of study participants by sex.

**Table 1:** Characteristic of the study population by socio-demographic, selected medical history, physical exam and biochemical tests

**Variable**	**Men (n=965)**	**Women (n=1138)**
Socio-demographic		
Age groups		
40-49	344 (35.6%)	396 (34.8%)
50-59	327 (33.9%)	401 (35.2%)
60-69	261 (27.0%)	322 (28.3%)
70-79	33 (3.4%)	19 (1.7%)
Place of birth		
Yazd province	845 (90.6%)	992 (90.1%)
Other provinces in Iran	88 (9.4%)	109 (9.9%)
Education		
Primary school and less	265 (27.7%)	553 (49.2%)
High school	316 (33.1%)	389 (34.6%)
Diploma and Graduate Diploma	249 (26.0%)	150 (13.3%)
BSc and more	126 (13.2%)	33 (2.9%)
Smoker (tobacco or hookah)	197 (20.6%)	33 (2.9%)
Past Medical History		
Diabetes mellitus	182 (19.0%)	242 (21.4%)
Hypertension	198 (20.8%)	337 (30.0%)
Hypercholesterolemia	181 (19.0%)	334 (29.5%)
Physical exam		
Waist circumference(cm)	96.2±12.1	97.7±12.0
BMI(kg/m^2^)	26.8±4.1	29.6±4.7
Overweight	439 (45.7%)	449 (39.6%)
Obese	200 (20.8%)	504 (44.5%)
Systolic blood pressure (mm Hg)	131.8±19.1	130.9±18.3
Diastolic blood pressure (mm Hg)	84.1±13.1	81.0±11.6
Laboratory exam		
Fasting blood sugar (mg/dL)	111.8±4.3	113.4±4.0
Triglyceride (mg/dL)	182.2±1.2	164.7±8.8
Total cholesterol (mg/dL)	198.9±42.9	207.0±44.2
HDL (mg/dL)	45.8±9.0	52.7±1.5
LDL (mg/dL)	116.9±3.7	121.7±3.9

WHO-EMR B model showed the highest proportion of low CVD risk predicted at 10 years, with a prevalence of >80%: while FRS had the highest prevalence of high CVD risk relative to other scores (more than 4 times). Table 2 shows the distribution of the risk group’s classification by each risk tool.

**Table 2:** Cardiovascular risk stratification in the 40-79 years age group according to different scores

**Risk classification**	**Risk Score**					
	**Persons classified byFRS** ^a^ **(n:2083)**		**Persons classified byIraPEN** ^b^ **(n:2070)**		**Persons classified byWHO** ^c^ **(n:2058)**	
	**Number**	**%**	**Number**	**%**	**Number**	**%**
Low	638	30.6	1781	86	1762	83.8
Moderate	887	42.2	163	7.8	207	9.8
High	558	26.5	126	6.1	89	4.2

^a^ FRS: Framingham Risk Score (office based); ^b^ IraPEN: WHO-EMR B risk score (laboratory-based); ^c^ WHO: WHO-EMR B risk score (non-laboratory based).

**Figure 1:**
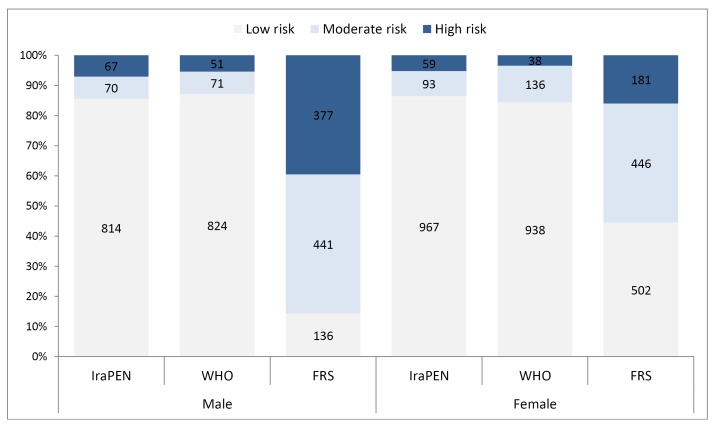
Percentage of cardiovascular risk classified according to different risk score tools by sex.

Figure 1 shows a 10-years risk estimate for CVD with various tools by sex. Compared to women, the Framingham model, unlike WHO-EMR B tools, estimates a higher percentage of men at risk. A slight agreement was observed between FRS model and WHO models, with 52.1% (IraPEN vs. FRS) and 51.3% (WHO vs. FRS) in women, compared to (21.3% and 19.6%) in men. Thus the agreements between tools were better in women. There is a high concordance between these three tools for categorizing individuals, both sexes, in the low-risk groups (above 90%), but there is a low agreement among them in classifying individuals at moderate/high-risk group. Discrepancy between FRS and IraPEN in categorizing people at risk were 55.5%, (McNemar, *P* < 0.0001) but this was not significant between IraPEN and non-laboratory WHO-EMR B scores (McNemar, *P* < 0.523; discrepancies, 5.8%).

**Figure 2:**
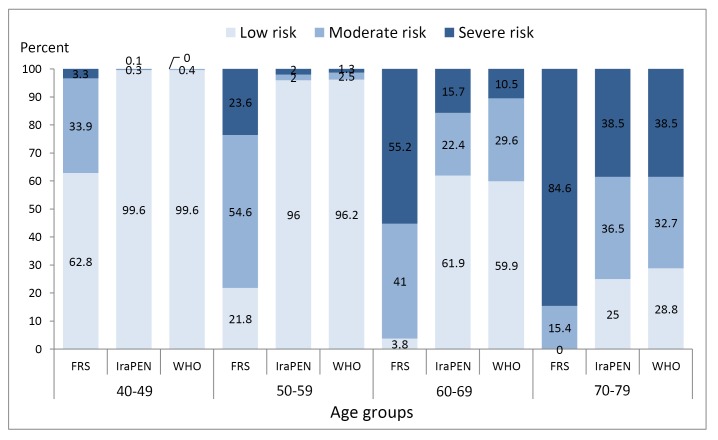
Percentage of adults’ cardiovascular risk classified according to different risk scores tools by age groups.

Data analysis based on age groups shows the difference between these two tools. With tool B, 23.6% of 50-60 years old were in high-risk groups; but the tool used in the national Iranian CVD scoring program (IraPEN based on WHO chart) only categorizes 2% of people at high risk (Figure 2).

64.5% of people (n=1145/1776) classified in the low-risk group with the recommended method (IraPEN), who were in the moderate and high-risk groups according to the FRS. Agreement between CVD risk scores was evaluated using Cohen’s weighted kappa between categories of predicted high risk. Agreement between the FRS and the IraPEN model was 37.9% (weighted kappa 0.17, *P* < 0.0001), in other words, a slight agreement was observed. The highest agreement was observed between laboratory base and non-laboratory base WHO-EMR B risk score (92.6%). The agreement between scores, overall and by sex can be seen in Table 3.

**Table 3:** Agreement between various CVD risk scores according to risk groups and sex

**Agreement**	**FRS vs.IraPENRisk Score** **No. (%)**	**FRS vs. WHO Risk Score** **No. (%)**	**IraPENvs. WHO Risk Score** **No. (%)**
Male	202 (21.3%)	185 (19.6%)	899 (95%)
Linear weighted kappa (95% CI)	0.107 (0.086-0.128)	0.086 (0.067-0.104)	0.844 (0.800-0.888)
*P* value*	<0.0001	0.035	<0.0001
Kappa rating	Slight	Slight	Perfect
Female	581 (52.1%)	596 (51.3%)	1007 (90.5%)
Linear weighted kappa (95% CI)	0.257 (0.219-0.295)	0.266 (0.230-0.302)	0.696 (0.638-0.753)
*P* value*	<0.0001	<0.0001	<0.0001
Kappa rating	Fair	Fair	Substantial
Total	783 (37.9%)	754 (36.8%)	1906 (92.6%)
Linear weighted kappa (95% CI)	0.174(0.153-0.195)	0.160 (0.141-0.180)	0.766 (0.729-0.803)
*P* value	<0.0001	<0.0001	<0.0001
Kappa Rating	Slight	Slight	Substantial

*Level of statistical significance (*P* < 0.05)

## Discussion

The classification of people at risk for CVD and its impact on the selection of preventive interventions is a necessity, but it is a complex task, due to the role of multiple risk factors associated with the onset of CVD. There is currently no specific tool developed, especially in Iran; thus, international scores are being used. Using the WHO chart in Iran’s health system nationwide, which is significantly different from that of the FRS, underestimated individuals at risk of CVD.

In our study, the agreement between risk classifications by different scores was generally slight. Overall, this difference in the classification of individuals in previous studies in different regions has been reported between risk scores and is consistent with our findings. For example, in the studies on Asian populations, the number of peoples classified at low, moderate or high risk, differed by applying different scores (FRS, SCORE and WHO/ISH).^10,21^ In these studies, the WHO chart, compared with the Framingham model, has underestimated ten-year cardiovascular risk by categorizing more people in the low-risk group. These results are similar to the present study, indicate that the use of the WHO chart for risk assessment is not advisable in all developing countries without their own score.^22^ However, some studies recommended the use of WHO’s charts in low-income countries ^11,23-25^ this recommendation is not consistent with our finding. Although all risk assessment tools do the same method, different tools, for one person, perform different categories. Ethnicity, socioeconomic status, and genetic of individuals in different geographic regions can be effective in increasing or decreasing the calculation of cardiovascular risk estimate, thus making the results of risk assessments different from ignoring these factors. The development and updating of well-known CVD risk scores also emphasizes the need to consider differences even within a population.

As far as we know, this study is the first published review of the WHO cardiovascular risk assessment tool with the Framingham method in Iran. However, others in different regions have also found that the majority of people are in the low-risk group.^26-28^ It should also be noted that some studies have reported that the Framingham tool has a higher estimate than other risk scores for high-risk groups.^29,30^ The discrepancy between low WHO/ISH (IraPEN) prediction and high Framingham score prediction is harmful to make CVD prevention and control programs uncertain. High-risk individuals are not being treated with prophylactic medication or may receive unnecessary intervention at low risk. Therefore, the use of risk-score tools without validation in a country/region may delay proper intervention or waste the resources.^31^

The current analysis demands the attention of both practitioners and health policymakers. As a result, risk assessment charts are specific to each population and cannot be used in different people. Hence, this seems necessary for the development of national CVD risk score for Iranian population depends on local risk factors measured. Until the design of a national instrument, it appears that replacing the WHO non-laboratory chart with Laboratory–bases in the health system would be more efficient. Its updated version can achieve a lower cost and a higher level of coverage to identify more high-risk individuals. Calibrating an international tool is another recommendation for designing national instruments.

This study has some strengths and weaknesses. This study used data from a community-based study with large sample size. There is no limitation in the detailed analysis between subgroups; however, the cross-sectional method of the study does not allow a comparison of the incidence of CVD with its risk prediction. The study generated baseline data at the recruitment phase and in the next phases of the study; it may be able to answer this important question. Both Laboratory and non-laboratory versions of the WHO/ISH tool were used in parallel, on an Iranian population for the first time and compared some popular risk assessment tools i.e., Framingham and WHO scores.

It should be noted that the Framingham tool considers a wide range of cardiovascular outcomes, but the WHO model mainly estimates the outcome of MI and stroke.^9^ the other difference between these tools is to consider/not to mention the previous history of CVD in risk scoring. These are classified in the high-risk group in the IraPEN model, while Framingham estimates the risk for people without a past history. Thus to compare these two tools, this should be considered.

## Conclusion

In IraPEN score, most of the people classified in the low-risk group and prevention is not recommended for them. So, for the identification of high CVD risk groups in Iranian populations, this risk assessment model is not useful. Our study shows that the accuracy and validity of risk assessment tools should be assessed before integrating it into primary health care. Slighted agreement level between these CVD risk stratification tools, both under- or over-estimation of CVD is harmful. It should be considered by the country’s health policymakers, as well as the urgent need for using alternative tools for the low-risk group.

The validation study recommended examining the international cardiovascular risk prediction models in a large-scale cohort. Prospective studies need to determine the contribution of each risk factor to the incidence of 10 years CVD in the Iranian population. It is necessary to enable the development of a national CVD risk score calculator.

## Competing interests

Authors declare no competing interests.

## Ethical approval

Ethics approval was obtained from the Ethics Committee of Shahid Sadoughi University of Medical Sciences (IR.SSU.MEDICINE.1396.311). All procedures performed in this study were approved. Written informed consent was obtained from all participants.

## Funding

This work was funded by Shahid Sadoughi University of Medical Sciences.

## Acknowledgments

We thank the people of Yazd for their participation in the Yazd Health study and Yazd Health workers for their help and support.
